# 3-(Diphenyl­methyl­idene)indolin-2-one

**DOI:** 10.1107/S1600536811028467

**Published:** 2011-07-23

**Authors:** Maosen Yuan, Qi Wang, Shujun Zhang, Peng Gao, Junru Wang

**Affiliations:** a College of Science, Northwest A&F University, Yangling 712100, Shannxi Province, People’s Republic of China

## Abstract

The title mol­ecule, C_21_H_15_NO, has an indoline-2-one and two benzene substituent groups which are arranged in a propeller-like fashion around the central C atom. The dihedral angle between the two benzene rings is 73.32 (16)° and those between the benzene rings and the indoline-2-one group are 76.54 (14) and 67.69 (14)°. In the crystal, there is an inter­molecular N—H⋯O hydrogen-bonding inter­action, which links the mol­ecules into chains extending along *c*.

## Related literature

For general background to indoline-2-one and its derivatives, see: Colgan *et al.* (1996 ▶). For the use of indoline-2-one as a precursor for the synthesis of organic luminescent mol­ecules, see: Ji *et al.* (2010 ▶). For a related structure, see: Spencer *et al.* (2010 ▶).
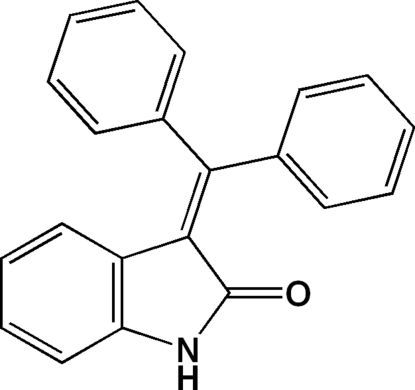


## Experimental

### 

#### Crystal data

C_21_H_15_NO
*M* *_r_* = 297.34
Orthorhombic, 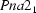

*a* = 11.0679 (11) Å
*b* = 17.6465 (16) Å
*c* = 7.8835 (6) Å
*V* = 1539.7 (2) Å^3^
*Z* = 4
Mo *K*α radiation
μ = 0.08 mm^−1^
*T* = 298 K
0.46 × 0.40 × 0.38 mm

#### Data collection

Bruker SMART CCD area-detector diffractometer
Absorption correction: multi-scan (*SADABS*; Sheldrick, 1996 ▶) *T*_min_ = 0.965, *T*_max_ = 0.971
9094 measured reflections
2072 independent reflections
1105 reflections with *I* > 2σ(*I*)
*R*_int_ = 0.050

#### Refinement

*R*[*F* ^2^ > 2σ(*F* ^2^)] = 0.037
*wR*(*F* ^2^) = 0.097
*S* = 1.06
2072 reflections
208 parameters
1 restraint
H-atom parameters constrained
Δρ_max_ = 0.15 e Å^−3^
Δρ_min_ = −0.15 e Å^−3^

### 

Data collection: *SMART* (Bruker, 2001 ▶); cell refinement: *SAINT* (Bruker, 2001 ▶); data reduction: *SAINT*; program(s) used to solve structure: *SHELXS97* (Sheldrick, 2008 ▶); program(s) used to refine structure: *SHELXL97* (Sheldrick, 2008 ▶); molecular graphics: *SHELXTL* (Sheldrick, 2008 ▶); software used to prepare material for publication: *SHELXTL*.

## Supplementary Material

Crystal structure: contains datablock(s) global, I. DOI: 10.1107/S1600536811028467/zs2123sup1.cif

Structure factors: contains datablock(s) I. DOI: 10.1107/S1600536811028467/zs2123Isup2.hkl

Supplementary material file. DOI: 10.1107/S1600536811028467/zs2123Isup3.cml

Additional supplementary materials:  crystallographic information; 3D view; checkCIF report

## Figures and Tables

**Table 1:** Hydrogen-bond geometry (Å, °)

*D*—H⋯*A*	*D*—H	H⋯*A*	*D*⋯*A*	*D*—H⋯*A*
N1—H1⋯O1^i^	0.86	2.23	2.974 (3)	144

Symmetry code: (i) 
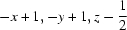
.

**Fig. 1.**
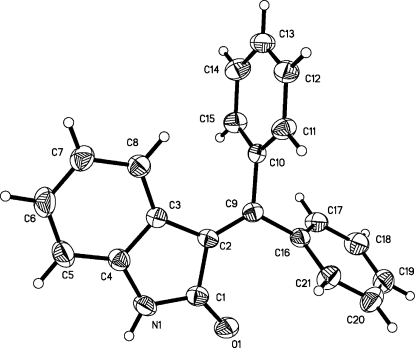
The molecular structure of the title compound with the atom numbering scheme. Displacement ellipsoids are drawn at the 30% probability level.

**Fig. 2.**
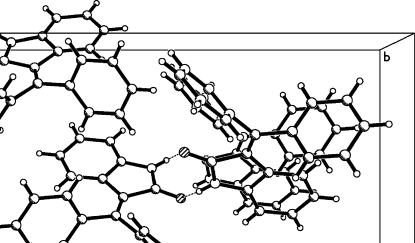
The molecular packing of (I) viewed along the c axis, with hydrogen bonds shown as dashed lines.

## supplementary crystallographic information

### Comment

Indoline-2-one and its derivatives are very important compounds as materials for the synthesis of pharmaceuticals (Colgan *et al.*, 1996). Indoline-2-one may also be used as a precursor for synthesizing organic luminescent molecules because of its perfect conformation (Ji *et al.*, 2010). In the course of exploring new electro-optic compounds, we obtained a intermediate compound C_21_H_15_NO (I) and the synthesis and structure are reported here.

The title compound has three substituent ring systems, an indoline-2-one ring and two benzene rings which are arranged in a propeller-like fashion around the central atom C9 (Fig. 1). The interplanar dihedral angle between the two benzene rings defined by C10–C15 and C16–C21 is 73.32 (16)°. The interplanar angles between these benzene planes and that of the indoline moiety are 76.54 (14)° and 67.69 (14)°, respectively. The molecules of (I) crystallize in the space group *Pna*2_1_ which is different from that of 3-(propan-2-ylidene)indolin-2-one (*P*-1) (Spencer *et al.* 2010). In the crystal structure there is an intermolecular N—H···O hydrogen-bonding interaction (Table 1) linking the molecules into one-dimensional chains which extend along *c* in the unit cell (Fig. 2).

### Experimental

Indolin-2-one (0.50 g, 3.76 mmol) was dissolved in THF (20 mL) and KOH (0.80 g, 14.3 mmol) was slowly added. After heating the stirred mixture at reflux temperature for 30 min, a solution of benzophenone (0.80 g, 4.40 mmol) in THF was slowly added and the refluxing continued for 2 h. The mixture was then cooled to 333 K and poured into water (200 mL) and was extracted with chloroform and dried over Na_2_SO_4_. After removing the solvent, the crude product was purified by column chromatography on silica gel, affording the title compound (yield: 0.28 g, 25%). The compound was then dissolved in THF and yellow crystals were formed on slow evaporation at room temperature over one week.

### Refinement

All H atoms were placed in geometrically calculated positions and refined using a riding model with C—H = 0.93 Å and N—H = 0.86 Å and with *U*_iso_(H) = 1.2*U*_eq_(C, N). Friedel pairs (1153) were merged for the data used in the refinement.

### Crystal data

**Table:**

C_21_H_15_NO	*F*(000) = 624
*M**_r_* = 297.34	*D*_x_ = 1.283 Mg m^−^^3^
Orthorhombic, *P**n**a*2_1_	Mo *K*α radiation, λ = 0.71073 Å
Hall symbol: P 2c -2n	Cell parameters from 1702 reflections
*a* = 11.0679 (11) Å	θ = 2.8–21.0°
*b* = 17.6465 (16) Å	µ = 0.08 mm^−^^1^
*c* = 7.8835 (6) Å	*T* = 298 K
*V* = 1539.7 (2) Å^3^	Block, yellow
*Z* = 4	0.46 × 0.40 × 0.38 mm

### Data collection

**Table:**

Bruker SMART CCD area-detector diffractometer	2072 independent reflections
Radiation source: fine-focus sealed tube	1105 reflections with *I* > 2σ(*I*)
graphite	*R*_int_ = 0.050
φ and ω scans	θ_max_ = 28.5°, θ_min_ = 2.8°
Absorption correction: multi-scan (*SADABS*; Sheldrick, 1996)	*h* = −14→14
*T*_min_ = 0.965, *T*_max_ = 0.971	*k* = −23→15
9094 measured reflections	*l* = −9→10

### Refinement

**Table:**

Refinement on *F*^2^	Primary atom site location: structure-invariant direct methods
Least-squares matrix: full	Secondary atom site location: difference Fourier map
*R*[*F*^2^ > 2σ(*F*^2^)] = 0.037	Hydrogen site location: inferred from neighbouring sites
*wR*(*F*^2^) = 0.097	H-atom parameters constrained
*S* = 1.06	*w* = 1/[σ^2^(*F*_o_^2^) + (0.0323*P*)^2^ + 0.1729*P*] where *P* = (*F*_o_^2^ + 2*F*_c_^2^)/3
2072 reflections	(Δ/σ)_max_ < 0.001
208 parameters	Δρ_max_ = 0.15 e Å^−^^3^
1 restraint	Δρ_min_ = −0.15 e Å^−^^3^

### Special details

**Table:**

Geometry. All esds (except the esd in the dihedral angle between two l.s. planes) are estimated using the full covariance matrix. The cell esds are taken into account individually in the estimation of esds in distances, angles and torsion angles; correlations between esds in cell parameters are only used when they are defined by crystal symmetry. An approximate (isotropic) treatment of cell esds is used for estimating esds involving l.s. planes.
Refinement. Refinement of F^2^ against ALL reflections. The weighted R-factor wR and goodness of fit S are based on F^2^, conventional R-factors R are based on F, with F set to zero for negative F^2^. The threshold expression of F^2^ > 2sigma(F^2^) is used only for calculating R-factors(gt) etc. and is not relevant to the choice of reflections for refinement. R-factors based on F^2^ are statistically about twice as large as those based on F, and R- factors based on ALL data will be even larger.

### Fractional atomic coordinates and isotropic or equivalent isotropic displacement parameters (Å^2^)

**Table:**

	*x*	*y*	*z*	*U*_iso_*/*U*_eq_	
N1	0.4749 (2)	0.42487 (13)	0.0945 (4)	0.0564 (7)	
H1	0.4439	0.4611	0.0361	0.068*	
O1	0.5893 (2)	0.49677 (11)	0.2730 (3)	0.0631 (6)	
C1	0.5549 (3)	0.43506 (16)	0.2223 (4)	0.0481 (8)	
C2	0.5846 (3)	0.35719 (15)	0.2880 (4)	0.0415 (7)	
C3	0.5159 (2)	0.30486 (15)	0.1807 (4)	0.0424 (7)	
C4	0.4489 (3)	0.34850 (16)	0.0692 (4)	0.0500 (8)	
C5	0.3681 (3)	0.3183 (2)	−0.0444 (5)	0.0695 (10)	
H5	0.3233	0.3490	−0.1167	0.083*	
C6	0.3564 (3)	0.2406 (2)	−0.0468 (5)	0.0728 (11)	
H6	0.3012	0.2184	−0.1206	0.087*	
C7	0.4244 (3)	0.19516 (18)	0.0577 (5)	0.0612 (9)	
H7	0.4163	0.1428	0.0513	0.073*	
C8	0.5051 (3)	0.22649 (16)	0.1725 (5)	0.0523 (8)	
H8	0.5511	0.1956	0.2428	0.063*	
C9	0.6510 (2)	0.34259 (15)	0.4264 (4)	0.0432 (7)	
C10	0.6611 (2)	0.26418 (15)	0.4946 (4)	0.0413 (7)	
C11	0.7606 (3)	0.22020 (16)	0.4565 (4)	0.0549 (9)	
H11	0.8228	0.2403	0.3912	0.066*	
C12	0.7683 (3)	0.14681 (17)	0.5144 (5)	0.0624 (10)	
H12	0.8355	0.1176	0.4873	0.075*	
C13	0.6788 (3)	0.11674 (17)	0.6108 (5)	0.0607 (9)	
H13	0.6844	0.0671	0.6496	0.073*	
C14	0.5802 (3)	0.15989 (18)	0.6504 (5)	0.0643 (10)	
H14	0.5187	0.1393	0.7162	0.077*	
C15	0.5712 (3)	0.23353 (16)	0.5939 (4)	0.0553 (8)	
H15	0.5042	0.2626	0.6230	0.066*	
C16	0.7198 (3)	0.40090 (16)	0.5234 (4)	0.0458 (8)	
C17	0.6978 (3)	0.41196 (17)	0.6938 (4)	0.0562 (9)	
H17	0.6395	0.3828	0.7480	0.067*	
C18	0.7609 (4)	0.46540 (19)	0.7850 (5)	0.0704 (10)	
H18	0.7447	0.4725	0.8997	0.085*	
C19	0.8472 (3)	0.50795 (19)	0.7065 (6)	0.0702 (11)	
H19	0.8891	0.5445	0.7677	0.084*	
C20	0.8726 (3)	0.49722 (19)	0.5391 (6)	0.0711 (11)	
H20	0.9326	0.5258	0.4868	0.085*	
C21	0.8093 (3)	0.44404 (17)	0.4473 (5)	0.0611 (9)	
H21	0.8267	0.4370	0.3330	0.073*	

### Atomic displacement parameters (Å^2^)

**Table:**

	*U*^11^	*U*^22^	*U*^33^	*U*^12^	*U*^13^	*U*^23^
N1	0.0730 (17)	0.0451 (15)	0.0510 (16)	0.0083 (12)	−0.0133 (16)	0.0055 (14)
O1	0.0791 (14)	0.0400 (12)	0.0703 (16)	−0.0001 (10)	−0.0080 (14)	−0.0016 (12)
C1	0.0563 (18)	0.0438 (17)	0.044 (2)	0.0028 (15)	0.0026 (16)	−0.0005 (16)
C2	0.0490 (15)	0.0380 (15)	0.0377 (16)	0.0044 (13)	0.0007 (14)	0.0011 (14)
C3	0.0458 (16)	0.0428 (16)	0.0385 (16)	0.0023 (13)	0.0028 (14)	−0.0016 (15)
C4	0.0557 (17)	0.0495 (18)	0.0448 (19)	0.0039 (15)	−0.0030 (15)	−0.0006 (16)
C5	0.083 (2)	0.072 (2)	0.054 (2)	0.005 (2)	−0.024 (2)	−0.002 (2)
C6	0.081 (2)	0.079 (3)	0.059 (2)	−0.006 (2)	−0.020 (2)	−0.018 (2)
C7	0.073 (2)	0.0549 (19)	0.056 (2)	−0.0049 (18)	−0.0027 (19)	−0.0126 (19)
C8	0.0607 (18)	0.0460 (17)	0.0503 (19)	0.0034 (15)	−0.0020 (17)	−0.0053 (16)
C9	0.0449 (16)	0.0387 (16)	0.0459 (18)	0.0038 (12)	0.0034 (15)	−0.0024 (14)
C10	0.0434 (15)	0.0387 (15)	0.0418 (18)	−0.0022 (13)	−0.0061 (15)	−0.0008 (14)
C11	0.0485 (17)	0.0464 (17)	0.070 (2)	−0.0011 (14)	0.0129 (18)	0.0042 (18)
C12	0.0601 (19)	0.0455 (19)	0.082 (3)	0.0089 (16)	−0.003 (2)	−0.0042 (18)
C13	0.084 (2)	0.0418 (18)	0.057 (2)	−0.0001 (18)	−0.009 (2)	0.0046 (18)
C14	0.075 (2)	0.056 (2)	0.062 (2)	−0.0100 (18)	0.012 (2)	0.0052 (18)
C15	0.0547 (17)	0.0511 (18)	0.060 (2)	0.0013 (15)	0.0083 (18)	0.0024 (18)
C16	0.0508 (16)	0.0371 (17)	0.050 (2)	0.0042 (14)	−0.0058 (16)	−0.0019 (14)
C17	0.072 (2)	0.0472 (19)	0.049 (2)	0.0000 (16)	−0.0001 (18)	0.0011 (16)
C18	0.101 (3)	0.053 (2)	0.057 (2)	0.001 (2)	−0.014 (2)	−0.0124 (19)
C19	0.078 (2)	0.050 (2)	0.083 (3)	−0.0047 (18)	−0.027 (2)	−0.015 (2)
C20	0.067 (2)	0.060 (2)	0.086 (3)	−0.0166 (18)	−0.004 (2)	−0.010 (2)
C21	0.065 (2)	0.060 (2)	0.058 (2)	−0.0122 (17)	0.0040 (19)	−0.0051 (18)

### Geometric parameters (Å, °)

**Table:**

N1—C1	1.353 (4)	C11—C12	1.376 (4)
N1—C4	1.392 (4)	C11—H11	0.9300
N1—H1	0.8600	C12—C13	1.356 (4)
O1—C1	1.221 (3)	C12—H12	0.9300
C1—C2	1.505 (4)	C13—C14	1.366 (4)
C2—C9	1.341 (4)	C13—H13	0.9300
C2—C3	1.465 (4)	C14—C15	1.377 (4)
C3—C4	1.384 (4)	C14—H14	0.9300
C3—C8	1.390 (4)	C15—H15	0.9300
C4—C5	1.374 (4)	C16—C17	1.379 (4)
C5—C6	1.377 (5)	C16—C21	1.386 (4)
C5—H5	0.9300	C17—C18	1.376 (4)
C6—C7	1.374 (5)	C17—H17	0.9300
C6—H6	0.9300	C18—C19	1.364 (5)
C7—C8	1.386 (4)	C18—H18	0.9300
C7—H7	0.9300	C19—C20	1.363 (6)
C8—H8	0.9300	C19—H19	0.9300
C9—C10	1.489 (4)	C20—C21	1.377 (5)
C9—C16	1.491 (4)	C20—H20	0.9300
C10—C15	1.377 (4)	C21—H21	0.9300
C10—C11	1.380 (4)		
			
C1—N1—C4	111.7 (2)	C12—C11—C10	120.4 (3)
C1—N1—H1	124.1	C12—C11—H11	119.8
C4—N1—H1	124.1	C10—C11—H11	119.8
O1—C1—N1	124.5 (3)	C13—C12—C11	120.6 (3)
O1—C1—C2	129.3 (3)	C13—C12—H12	119.7
N1—C1—C2	106.1 (3)	C11—C12—H12	119.7
C9—C2—C3	129.3 (2)	C12—C13—C14	119.5 (3)
C9—C2—C1	125.1 (3)	C12—C13—H13	120.2
C3—C2—C1	105.3 (2)	C14—C13—H13	120.2
C4—C3—C8	118.6 (3)	C13—C14—C15	120.7 (3)
C4—C3—C2	107.1 (2)	C13—C14—H14	119.7
C8—C3—C2	134.4 (3)	C15—C14—H14	119.7
C5—C4—C3	123.2 (3)	C10—C15—C14	120.1 (3)
C5—C4—N1	127.1 (3)	C10—C15—H15	119.9
C3—C4—N1	109.7 (3)	C14—C15—H15	119.9
C4—C5—C6	117.2 (3)	C17—C16—C21	118.0 (3)
C4—C5—H5	121.4	C17—C16—C9	120.5 (3)
C6—C5—H5	121.4	C21—C16—C9	121.5 (3)
C7—C6—C5	121.4 (3)	C18—C17—C16	121.1 (3)
C7—C6—H6	119.3	C18—C17—H17	119.4
C5—C6—H6	119.3	C16—C17—H17	119.4
C6—C7—C8	120.8 (3)	C19—C18—C17	119.7 (4)
C6—C7—H7	119.6	C19—C18—H18	120.1
C8—C7—H7	119.6	C17—C18—H18	120.1
C7—C8—C3	118.9 (3)	C20—C19—C18	120.5 (4)
C7—C8—H8	120.6	C20—C19—H19	119.8
C3—C8—H8	120.6	C18—C19—H19	119.8
C2—C9—C10	120.9 (3)	C19—C20—C21	119.9 (4)
C2—C9—C16	124.4 (3)	C19—C20—H20	120.0
C10—C9—C16	114.7 (3)	C21—C20—H20	120.0
C15—C10—C11	118.7 (3)	C20—C21—C16	120.7 (4)
C15—C10—C9	121.1 (2)	C20—C21—H21	119.6
C11—C10—C9	120.3 (3)	C16—C21—H21	119.6
			
C4—N1—C1—O1	−177.5 (3)	C1—C2—C9—C16	−8.8 (4)
C4—N1—C1—C2	−0.1 (3)	C2—C9—C10—C15	−80.4 (4)
O1—C1—C2—C9	4.6 (5)	C16—C9—C10—C15	99.8 (3)
N1—C1—C2—C9	−172.6 (3)	C2—C9—C10—C11	98.5 (3)
O1—C1—C2—C3	178.8 (3)	C16—C9—C10—C11	−81.3 (3)
N1—C1—C2—C3	1.6 (3)	C15—C10—C11—C12	1.1 (4)
C9—C2—C3—C4	171.4 (3)	C9—C10—C11—C12	−177.8 (3)
C1—C2—C3—C4	−2.5 (3)	C10—C11—C12—C13	−0.5 (5)
C9—C2—C3—C8	−7.5 (6)	C11—C12—C13—C14	0.0 (5)
C1—C2—C3—C8	178.6 (3)	C12—C13—C14—C15	−0.2 (5)
C8—C3—C4—C5	2.9 (5)	C11—C10—C15—C14	−1.3 (4)
C2—C3—C4—C5	−176.2 (3)	C9—C10—C15—C14	177.5 (3)
C8—C3—C4—N1	−178.4 (3)	C13—C14—C15—C10	0.9 (5)
C2—C3—C4—N1	2.5 (3)	C2—C9—C16—C17	122.6 (3)
C1—N1—C4—C5	177.1 (3)	C10—C9—C16—C17	−57.6 (4)
C1—N1—C4—C3	−1.6 (4)	C2—C9—C16—C21	−59.1 (4)
C3—C4—C5—C6	−1.1 (5)	C10—C9—C16—C21	120.6 (3)
N1—C4—C5—C6	−179.5 (3)	C21—C16—C17—C18	1.5 (5)
C4—C5—C6—C7	−1.3 (6)	C9—C16—C17—C18	179.8 (3)
C5—C6—C7—C8	1.8 (6)	C16—C17—C18—C19	−0.5 (5)
C6—C7—C8—C3	0.1 (5)	C17—C18—C19—C20	−0.8 (6)
C4—C3—C8—C7	−2.4 (5)	C18—C19—C20—C21	1.2 (6)
C2—C3—C8—C7	176.4 (3)	C19—C20—C21—C16	−0.1 (5)
C3—C2—C9—C10	−1.3 (5)	C17—C16—C21—C20	−1.2 (5)
C1—C2—C9—C10	171.5 (3)	C9—C16—C21—C20	−179.5 (3)
C3—C2—C9—C16	178.4 (3)		

### Hydrogen-bond geometry (Å, °)

**Table:**

*D*—H···*A*	*D*—H	H···*A*	*D*···*A*	*D*—H···*A*
N1—H1···O1^i^	0.86	2.23	2.974 (3)	144

Symmetry codes: (i) −*x*+1, −*y*+1, *z*−1/2.
